# Base and Prime Editing in the Retina—From Preclinical Research toward Human Clinical Trials

**DOI:** 10.3390/ijms232012375

**Published:** 2022-10-16

**Authors:** Tiffany Yee, Katherine J. Wert

**Affiliations:** 1 Department of Ophthalmology, UT Southwestern Medical Center, Dallas, TX 75390, USA; 2 Peter O’Donnell Jr. Brain Institute, UT Southwestern Medical Center, Dallas, TX 75390, USA; 3 Department of Molecular Biology, UT Southwestern Medical Center, Dallas, TX 75390, USA; 4 Hamon Center for Regenerative Science and Medicine, UT Southwestern Medical Center, Dallas, TX 75390, USA

**Keywords:** CRISPR, gene editing, AAV, retina, retinal degeneration, gene therapy, photoreceptor degeneration

## Abstract

Inherited retinal diseases (IRDs) are a clinically and genetically heterogeneous group of diseases that are one of the leading causes of vision loss in young and aged individuals. IRDs are mainly caused by a loss of the post-mitotic photoreceptor neurons of the retina, or by the degeneration of the retinal pigment epithelium. Unfortunately, once these cells are damaged, it is irreversible and leads to permanent vision impairment. Thought to be previously incurable, gene therapy has been rapidly evolving to be a potential treatment to prevent further degeneration of the retina and preserve visual function. The development of clustered regularly interspaced short palindromic repeats (CRISPR)-CRISPR-associated protein 9 (Cas9) base and prime editors have increased the capabilities of the genome editing toolbox in recent years. Both base and prime editors evade the creation of double-stranded breaks in deoxyribonucleic acid (DNA) and the requirement of donor template of DNA for repair, which make them advantageous methods in developing clinical therapies. In addition, establishing a permanent edit within the genome could be better suited for patients with progressive degeneration. In this review, we will summarize published uses of successful base and prime editing in treating IRDs.

## 1. Introduction

**Figure 1:**
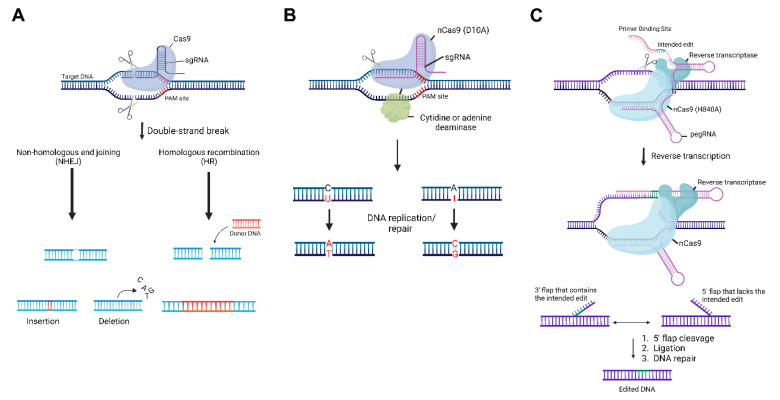
Overview of the clustered regularly interspaced short palindromic repeats (CRISPR) toolbox. (**A**) Illustration of the original CRISPR/CRISPR-associated protein 9 (Cas9) System. Upon recognition of the target sequence, Cas9 will cleave both strands of deoxyribonucleic acid (DNA). This will result in two natural DNA repair pathways—non-homologous end joining and homologous recombination, which will lead to a small insertion or deletion, or a large insertion containing a donor DNA template, respectively. (**B**) Illustration of the base editing system. A Cas9 nickase (nCas9) is fused to a cytidine or adenine deaminase and upon nicking the single strand of DNA, the enzyme will deaminate the cytosine or adenine, leading to a uracil or inosine, respectively. Then, DNA replication or repair will recognize the change and lead to a permanent base pair conversion. (**C**) Illustration of the prime editing system. Prime editing utilizes a Cas9 nickase fused to a reverse transcriptase and a prime editing guide ribonucleic acid (pegRNA) that contains the spacer sequence, primer binding site, and the template containing the intended edit. After recognition and the single-strand nick, the primer binding site will allow for the exposed 3′-hydroxyl end of the nicked DNA strand to initiate the reverse transcription of the template. This results in an intermediate that includes two DNA flaps: a 3′ flap that contains the desired edit, and a 5′ flap that contains the unedited strand. After equilibration between the two flaps, cleavage, ligation, and DNA repair, the stably edited DNA sequence remains. PAM, protospacer adjacent motif; sgRNA, single guide ribonucleic acid.

Since the advent of clustered regularly interspaced short palindromic repeats (CRISPR) editing technologies [1], there has been an emergence in its widespread application. CRISPR systems were originally found in bacteria and archaea to provide adaptive immunity against invasive nucleic acids, such as viruses and plasmids. Since then, the CRISPR system has been adapted to be used in mammalian cells for biomedical research purposes. To accomplish this, the deoxyribonucleic acid (DNA) endonuclease CRISPR-associated protein 9 (Cas9) can be directed by a guide ribonucleic acid (gRNA) for site-specific double-stranded DNA cleavage. Upon cleavage, natural DNA repair mechanisms result in either small insertions and deletions (called indels) introduced by non-homologous end joining, or insertion of a new DNA sequence by homology-directed repair [2] (Figure 1A). Indels are useful for the disruption of the genetic target of interest, while sequence insertion can be used to restore or alter gene function. In the original CRISPR system, Cas9 is guided by two separate RNA molecules—a CRISPR RNA (crRNA) that recognizes the site of interest through complementary base pairing, and a trans-activating CRISPR RNA (tracrRNA) that complexes with the crRNA to bind with Cas9. These two RNAs form a functional gRNA, which together with Cas9, recognize the target site. In addition, protospacer adjacent motif (PAM) sequences are short DNA sequences around 2–6 base pairs that serve as a binding signal for Cas9 to direct cleavage. In some cases, the PAM sequence can be limiting due to the dependency of its location to be nearby to the targeted edit site.

In recent years, advances in this CRISPR genome editing technology have been developed. For instance, in order to bypass the cellular toxicity that DNA double strand breaks can introduce, additional methods were devised that could alter genetic activity without cutting the DNA strand. First, a dead Cas9 (dCas9) [3] can be used in place of Cas9. dCas9 is a mutant form of Cas9 where the endonuclease activity is removed. Two methods using this dCas9 are CRISPR interference (CRISPRi) [3,4] and CRISPR activation (CRISPRa) [5]. CRISPRi and CRISPRa utilize the dCas9 to form a complex with the single guide RNA (sgRNA) that is fused with a transcription terminator derived from *Streptococcus pyogenes*. In CRISPRi, the dCas9 can be fused to a Krüppel-associated box (KRAB) repressor [6] to silence gene expression. CRISPRa [5] is like CRISPRi in that it uses a sgRNA to guide the dCas9 to its target, but instead fuses the dCas9 to transcriptional activators, such as VP64 or p65, to increase gene expression. Another two methods being used are termed the CRISPRon and CRISPRoff systems, acting as epigenetic editors [7]. In CRISPRon/CRISPRoff, the dCas9 can be used to establish DNA methylation, DNA demethylation, and repressive histone modifications. To accomplish this, the CRISPRon/off system fuses the dCas9 with ten-eleven translocation (TET) enzymes and p65-VP64 activators (CRISPRon), and DNA methyltransferases and KRAB domains (CRISPRoff), to epigenetically regulate gene expression, bypassing the cellular toxicity DNA double strand breaks can introduce [8]. Lastly, there was the development of the Cas9 nickase (nCas9) [9,10]. nCas9 is an additional variant of the Cas9 nuclease that differs by a point mutation (D10A or H840A) in the RuvC or HNH nuclease domain, respectively, that allows it to nick single stranded DNA as opposed to the Cas9 double stranded cleavage. This nCas9 has been incorporated into CRISPR gene editing for base and prime editing, as described further in this review.

## 2. Base Editors

Base editors are a class of genome editors that can achieve a targeted conversion of a single base pair (Figure 1B). There are two classes of base editors—cytosine base editors (CBEs) [11] and adenine base editors (ABEs) [12], which can convert C•G to T•A and A•T to G•C, respectively. In cytosine base editors, cytidine deaminases can be used to catalyze the deamination of cytosine, which leads to uracil, the RNA equivalent of thymine. This is accomplished as cytosine and uracil only differ by the presence of a methyl group, and the uracil is then read as thymine following DNA replication or repair, and transcription. This results in a permanent conversion to an A/T base pair instead of the original G/C pair. In ABEs, researchers took advantage of a naturally occurring chemical change that resulted from a spontaneous deamination of cytosine and modified this concept for deaminating adenine. The deamination of adenine yields inosine, which is recognized as guanine by polymerases. This results in a permanent conversion to a G/C base pair instead of the original A/T base pair. Another key component of CBEs and ABEs is the introduction of the D10A nCas9, which allows these base editors to introduce efficient and precise point mutations with less off-target genome modifications and without double-strand DNA breaks and homology-directed repair processes [11]. Recently, several efforts have been made to improve these base editors by increasing their editing window. These new modifications use alternative recognizable PAMs [13,14,15], and alternative Cas proteins such as Cas12 [14,16]. While these have increased the editing window [15], base editors do still have the potential for bystander effects [17] and are still only limited to 4 base pair conversions.

## 3. Prime Editors

Prime editing [18] is another novel genome editing method used to rewrite DNA without the use of double-stranded breaks or additional donor DNA templates. By using an RNA-programmable H840A *Streptococcus pyogenes* (Sp)Cas9 nickase fused to an engineered reverse transcriptase, as well as a prime editing guide RNA (pegRNA) that dually specifies the intended edit location and encodes the desired edit, prime editing has the potential to induce any small-sized genetic change—including insertions, deletions, and all twelve possible point mutations at a single base pair resolution. To accomplish this, the pegRNA forms a complex with the genomic DNA and with the spacer sequence (Figure 1C). This binds the target site and allows only the PAM-containing strand to be nicked by nCas9. When the targeted genomic site is nicked, a 3′-hydroxyl group is exposed and can be used to prime the reverse transcription of the extension sequence encoding the desired edit. This results in an intermediate containing a 3′ flap with the reversely transcribed edit, and a 5′ flap with the original non-edited sequence. Equilibration between the two flaps, cleavage, ligation, and naturally occurring DNA repair mechanisms will incorporate the desired editing outcome. In addition, prime editing evades the need for a PAM to be situated near the target site, which increases editing capabilities for areas of the genome that would otherwise not be able to be edited by the traditional CRISPR technologies [16,18].

However, there are still limitations to the prime editing technology. For instance, unprotected nuclear RNAs are susceptible to degradation. Thus, the 3′ extension of pegRNAs is exposed and more prone to be degraded, which can inhibit the ability to incorporate the target edit [19]. As a result, there have been advances made to optimize the pegRNAs and prevent the degradation of the 3′ extension. The development of the prime editor 3 (PE3) [18] and PE3b systems introduce a second gRNA that nicks the unedited strand after flap excision to increase preferential repair of the non-edited strand for improved editing efficiency. In addition, enhanced pegRNAs [19] have been generated that install a structured RNA pseudoknot at the 3′ end to protect it from exonucleases by increasing RNA stability. Overall, prime editing not only maintains the advantages of evading a double stranded break, but also increases versatility and allows for a broader editing range [20] by being able to install any single base-to-base change, delete at least 80 nucleotides, and insert at least 44 nucleotides [18,21]. 

## 4. Inherited Retinal Diseases (IRDs)

**Figure 2:**
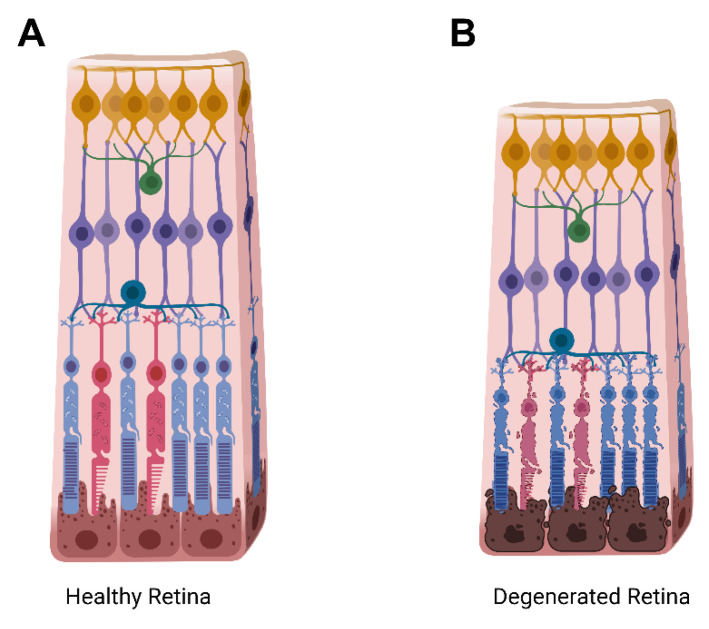
Cartoon schematic of a healthy retina in comparison to a degenerating retina. (**A**) Illustration of the retinal layers in an intact, healthy, retina. (**B**) Illustration of a degenerating retina with rod photoreceptors depicted in blue, and cone photoreceptors depicted in red. The total retinal thickness, as well as the outer nuclear layer containing the rods and cones thins upon degeneration as photoreceptor cells shrink, lose functionality, and die. Yellow, ganglion cells; green, amacrine cells; teal, horizontal cells; purple, bipolar cells; brown, retinal pigment epithelium.

These CRISPR-based technologies have allowed for scientists to rapidly accelerate research by creating cell lines and animal models for disease modeling, as well as testing potential gene therapies for clinical therapeutics. CRISPR-Cas9 editing has now been tested in several genetic diseases in various organ systems, ranging from blood diseases [22] to cancer [23]. Inherited retinal diseases (IRDs) are a clinically and genetically heterogeneous group of diseases that are one of the leading causes of vision loss in young and aged individuals. They are mainly caused by a loss of the post-mitotic photoreceptor neurons of the retina, or by the degeneration of the retinal pigment epithelium (RPE) (Figure 2). Unfortunately, once these cells undergo damage, it is irreversible and leads to permanent vision impairment. The time of onset, disease progression, and inheritance pattern can vary for IRDs, making treatment options complicated, and they are currently attributed to over 280 genes, resulting in a large amount of clinical heterogeneity [24,25]. Due to the accessibility, the anatomical structure, and the immune privileged state of the eye [21,25,26], treating IRDs using CRISPR technology is of special interest in the field of ophthalmology.

## 5. Gene Therapy for IRDs

**Figure 3:**
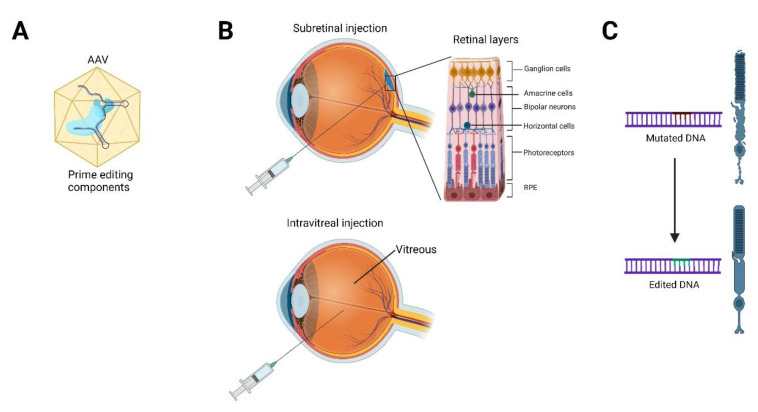
Schematic depicting the gene therapy delivery approach for inherited retinal diseases. (**A**) Gene editing components, such as prime editors, will be packaged into the delivery vector, such as an adeno-associated virus (AAV). (**B**) Subretinal delivery involves injection into the space between the retinal pigment epithelium (RPE) and photoreceptors to directly target those cells, while intravitreal injection delivers the viral vector into the vitreous body and can best target the inner retina, optic nerve, and lens. (**C**) After injection, gene editing will occur in the targeted cell (photoreceptor shown in image), and the mutated deoxyribonucleic acid (DNA) will be edited to keep the cell healthy and functional.

In recent years, gene therapy has been the focus for treating IRDs [27]. Commonly, the two routes of delivery are by subretinal or intravitreal injection, which maximizes transduction to the target cells in the eye (Figure 3). At the end of 2017, the United States of America Food and Drug Administration (FDA) approved Luxturna [28] (voretigene neparvovecrzyl) for the treatment of an IRD called Leber congenital amaurosis (LCA). This is the first in vivo gene therapy for IRDs, as well as the first in vivo gene therapy to be FDA approved. Luxturna acts by delivering a subretinal injection of a functional copy of *RPE65* packaged in an adeno-associated viral (AAV) vector to supplement the two mutant copies of *RPE65* present in LCA patients [29]. *RPE65* is expressed in the RPE and is critical for the regeneration of 11-*cis*-retinal from all-*trans*-retinal after photoreceptor activation by light.

## 6. Limitations in Gene Therapy for IRDs

While these *RPE65* gene therapy trials have shown tremendous promise for the efficacy of gene therapy supplementation approaches, there are still concerns and limitations present to be addressed. First, there is the potential for the exogenous transgene to be silenced over time [30,31], limiting the duration of treatment efficacy. Second, as most IRDs are progressive, little is known about gene therapy efficacy as patients age. Third, current gene therapy approaches supplement loss-of-function mutations with an additional copy of a gene, providing efficacy for recessive IRDs. However, treating patients with dominant IRD mutations requires a safe method for gene editing, or another therapeutic approach outside of gene therapy to be used.

In addition, the AAV vectors utilized to deliver the gene supplement carry their own limitations. For instance, the genes implicated in commonly inherited retinal degenerative diseases are not easily treated with AAV vectors because they are limited by its packaging size of 4.7 kb. Coding sequences often exceed the kilobase capacity of an AAV vector, including *ABCA4* in the IRD Stargardt disease, which is 6.8 kb [32]. Furthermore, AAV vectors can be genotoxic [33] even with their low rates of host genome integration. This possibility for the transgene to integrate into the host genome could lead to large-scale changes in the transcriptome, either through chromatin reorganization or disrupting neighboring genes and downstream pathways [33].

## 7. Base and Prime Editing for IRDs

Since many of the IRDs are monogenic and can be attributed to a single point mutation, base and prime editors hold great promise for gene editing without the high potential for off-target effects from the traditional CRISPR/Cas9 double-strand break methods [34]. In particular, split-intein [35] AAV delivery is promising for the use of delivering base and prime editors directly to the gene of interest to permanently correct the mutation. Since gene editors are generally too large to fit into a single AAV for delivery, studies have used dual-AAV approaches [36,37,38]. In these approaches, the base editor is divided into two halves, an N-terminal and C-terminal half. Each half is fused to a small *trans-*splicing intein where it will recombine and express the full-length base editor upon transduction into the target cell [36]. In fact, since these delivery approaches show signs of success, preclinical testing is now ongoing using base and prime editors to treat IRDs.

## 8. Base Editing in Leber Congenital Amaurosis (LCA)

Although it was developed recently, there have already been strides made in utilizing base editing as a potential gene therapy in IRDs to lead to long-term protection of vision. For example, the *rd12* mouse strain is a preclinical model for LCA, where the mice present with cone photoreceptor degeneration due to RPE65 deficiency [39,40]. Using an adenine base editor (ABE), Choi and Suh et al. show that an in vivo correction of an *Rpe65* mutation restored cone-mediated visual function and preserved cone survival in these *rd12* mice [39,40]. To improve upon their base editing efficiency after subretinal delivery to the *rd12* mice, they tested additional ABE variants with expanded PAM compatibility. They also tested various gRNAs, as well as the different ABE variants, in order to find the optimal base editing efficiency prior to testing in vivo in the preclinical *rd12* mouse model. Once they determined the optimal ABE variants and gRNAs, they performed a subretinal delivery of their gene editing components to distribute them into the space between the RPE and the photoreceptor cells [41]. This was achieved by injecting a single lentivirus vector containing the sgRNA and NG-ABE sequences into three-week-old *rd12* mice. Sequencing analysis showed an average of 54% A-to-G conversion at the target adenine, and an average of 27% of functionally rescued *Rpe65* alleles [39]. 

However, lentivirus can integrate into the host genome and is not the best delivery method for moving forward to clinical trials in terms of safety. They then tested delivery in the eye using an AAV serotype 2 (AAV2) [39]. The AAV delivery was successful, albeit they did not see phenotypic rescue until seven weeks after injection. Thus, AAV delivery required a longer time window before therapeutic efficacy in comparison to lentiviral delivery, where rescue was detected by three weeks post-injection. As mentioned previously, a main limitation of AAV vectors lie in its packaging constraints. To circumvent the packaging limitations, they had to use a dual AAV-mediated approach in which the ABE is divided and packaged as two separate AAV2 vectors. Once transduced, the ABE can be reconstituted and complexed with the sgRNA. While their base editing using AAV2 was still successful, the delay in phenotypic response could be due to the requirement of two vectors and the necessity for the base editing machinery to re-complex in the cell. However, the efficacy after seven weeks was promising, and this same group has now tested this approach in *rd12Gnat1^−/−^* mice, which render the mice to be cone-function dominant [39]. This mouse model was created by crossing the *rd12* mice with a *Gnat1^−/−^* strain, which lack the α subunit of rod transducin that is required for rod photoreceptor signal transduction. This allowed them to focus on the cone photoreceptors, and test whether their ABE approach would prevent cone degeneration and loss of function. Similarly, in this new study, they observed restoration of cone-mediated visual function and improved cone survival in base-edited mice, persisting six months post-treatment.

## 9. Base Editing in Retinitis Pigmentosa (RP)

Retinitis pigmentosa (RP) is the leading cause of progressive vision loss and inherited blindness, which affects approximately 1 in 4000 people worldwide [42]. RP is a genetically heterogeneous disease caused by mutations in more than sixty genes and follows autosomal recessive, autosomal dominant, and X-linked inheritance patterns [43]. While RP can be attributed to mutations in multiple genes, autosomal dominant RP [44] is largely linked to a mutation in the rhodopsin gene, which encodes the most abundant protein in the rod photoreceptor cells of the retina. Rhodopsin (*RHO*) plays a central role in the phototransduction pathway, and when mutated, leads to retinal dysfunction and degeneration of the photoreceptors in a rod-cone manner [45]. In Kaukonen et al. [45], all *RHO* variants were analyzed and separated based on variant type and accessible and nearby PAM sites. This provides a list of the *RHO* variants that can be targeted by gene editing approaches, such as base and prime editing. Currently, there have not been any published results on base editing of the rhodopsin gene, however, this is likely to be a future avenue of treatment for patients with autosomal dominant RP caused by mutations in *RHO*.

Additionally, the most common form of autosomal recessive RP is associated with mutations in *PDE6*, which encodes the rod cyclic guanosine monophosphate (cGMP)-phosphodiesterase, a key enzyme required in phototransduction to hydrolyze cGMP for channel closure [46]. In a recent bioRxiv paper [47], Yang et al. reported a base editing approach using an AAV-mediated ABE strategy to correct a *Pde6β* mutation in the photoreceptor cells. For this study, they used the *rd10* preclinical mouse model, which carries a mutation in *Pde6β* that causes an RP disease phenotype in the mouse. They performed subretinal delivery of AAV serotype 8 (AAV8)-ABE at two weeks of age to correct the *Pde6β* mutation with up to 37.41% efficiency at the DNA level. This restored PDE6β expression with up to 91.95% efficiency at the complementary (c)DNA level. PDE6*β* restoration in the treated mice was also validated by Western blot and immunolabeling experiments. The retinas of the treated mice showed both rod and cone cell preservation via immunostaining, almost comparable to those in wild type mice. They saw persistence of rod and cone rescue, as well as visual function, at twelve weeks of age. While this work is promising to protect against photoreceptor degeneration in patients with RP, this is only one gene of many, and more preclinical studies are needed to test the safety and efficacy of base editors for IRDs before moving forward into human clinical trials. 

## 10. Base Editing in Stargardt Macular Dystrophy

Stargardt macular dystrophy (STGD1) is the most common form of inherited childhood blindness worldwide with a prevalence of 1 in 8–10,000 individuals [48]. It is an autosomal recessive disease caused by mutations in *ABCA4*, the gene that codes for ATP-binding cassette transporter protein family member 4. *ABCA4* has a coding sequence length of 6.8 kB, which is too large for the standard AAV packaging capacity [32]. While groups have attempted an AAV dual vector strategy [32] as used for the LCA preclinical studies described previously, the editing efficiency could be improved by using a single base or prime editor. To look into this approach, Piotter et al. screened mutations in three available databases to reveal which of the approximately 1200 known pathogenic mutations in *ABCA4* are editable by targeted DNA base editing [48]. Further studies for the safety and efficacy of this approach are ongoing in the field of ophthalmology, and there is promise for the use of base and prime editing technology to treat this IRD.

## 11. Prime Editing in LCA

Prime editing, alongside base editing, is another potential therapeutic approach for gene editing to preserve vision in patients with IRDs. As a newer technique, studies are just arising that utilize prime editing in the retina. Similar to base editing in LCA, Jang et al. tested [49] an in vivo prime editing approach in the retinas of adult *rd12* mice, carrying a nonsense mutation caused by a C-to-T transition in the *Rpe65* gene. After testing various pegRNA efficiencies, they used AAV to deliver the prime editor 2 (PE2) with an mCherry reporter, as well as the pegRNA encoding the target sequence. Due to PE2 being 6273 bp, they used a *trans*-splicing AAV2 vector to package all of the components. *rd12* mice underwent subretinal injections—delivering two *trans*-splicing PE2-expressing AAVs, one encoding the N-terminal half of PE2 and the other encoding the C-terminal half, as well as a separate AAV containing the pegRNA and sgRNA—at three weeks of age. The treated mice were analyzed six weeks post-injection. Approximately 23% of the RPE was mCherry positive, reflecting the amount of the AAV2-PE2 that was able to transduce the RPE cells. Subsequent sequencing of the RPE cells showed an average prime editing efficiency range from 4.1% to 7.4%, with no detectable off-targets. Even with this lower efficiency rate, visual function was vastly improved in the injected mice as tested by electroretinography (ERG). Scotopic a- and b- wave amplitudes in treated mice were on average 59% and 27%, respectively, of their wild type counterparts, with great improvement over untreated *rd12* mice. These results are encouraging for the use of prime editors for clinical treatment of LCA and other IRDs. This study shows that prime editors can be effective, similar to base editors, in preserving visual function in preclinical models of LCA. All of these studies in models of IRDs highlight the excitement in the field of ophthalmology for the advent of base and prime editing technology, and the advancements already being made toward clinical therapeutics.

## 12. Discussion

Researchers have seen great success with the efficiency of both base and prime editing in somatic cells of mice, to the extent that it shows promise for future success in human patients [50,51]. However, there are still many challenges to overcome before these gene editing technologies will be available to treat human patients with IRDs. One challenge that is still being addressed is having an efficient gene editing system for areas of the genome that are difficult to access or target, as IRDs can be attributed to over 280 genes [34]. To target them all would be costly, unfeasible, and inefficient. Some genes in retinal diseases are not in locations that are PAM compatible, and some have mutations far too complex for base or prime editing. For example, choroideremia has yet to be used for base and prime editing applications. Choroideremia is an X-linked recessive disorder characterized by a frameshift mutation in the *CHM* gene, which encodes Rab escort protein 1 (REP-1) and is important for intracellular protein trafficking [52]. Patients with choroideremia are subjected to the slow degeneration of photoreceptors, RPE, and choroid, which is the vascular layer of the eye [52]. This manifests as peripheral visual field loss and night visual impairment. Since the mutation is not characterized by a single base pair mutation, base and prime editing technologies are not likely to be the best options as a treatment approach. However, since the *CHM* gene is only 1.9 kb, there have been studies showing progress in treating choroideremia using AAV gene therapy to enhance the transgene expression. 

In addition, retinal degeneration pathology cannot be wholly attributed to genetic mutations in coding regions. The need to understand mutations in the epigenetic landscape is crucial to moving forward with additional therapies and targets for human patients. While there are not many studies examining the effects of epigenetic mutations in IRDs [53], many epigenetic marks play a role in the expression and variability of retinal genes involved in phototransduction and development [54,55]. It is important to take into consideration the potential effects that DNA or histone methylation have on the pathogenesis of IRDs over time. In the future, it is possible to go beyond the scope of base and prime editing to develop and test epigenetic editors, such as CRISPRon or CRISPRoff systems, for the management of retinal gene expression during IRD progression.

Like with all genome editing technologies, the potential for off-target effects is a challenge that remains to be addressed in therapeutic contexts. Off-target effects are mutations resulting from aberrant cleavage at unintended target sites, as well as disruptions resulting from the intended edit [56]. Off-target effects can lead to genomic instability and disruptions in other genes that are otherwise functional. For instance, a limiting factor of base editors is the potential for “bystander” edits [57,58]. Nearby adenines or cytosines could be deaminated and affect the precision of the targeted editing outcome. In designing base editors, it is crucial for the variant to be able to discriminate between the desired edit and an undesired one. 

Furthermore, the introduction of any genome editing agent, whether as AAV [33] or lentivirus, has the potential for immunogenicity [59] and genotoxicity. While scientists are constantly optimizing delivery methods and increasing editing efficiency, the risk for immunotoxicity and genotoxicity post-editing still needs to be monitored upon progression into clinical trials. For example, a major concern with base and prime editing is the immunogenicity of Cas9 and its ability to induce an inflammatory response in the host [60]. The most widely used orthologs of Cas9 are *Staphylococcus aureus* (SaCas9) and *Streptococcus pyogenes* (SpCas9), bacterial species that are present and infect humans normally during life [61,62]. Charlesworth et al. analyzed human serum for the presence of anti-Cas9 antibodies and detected antibodies against both SaCas9 and SpCas9 in more than half of their donor serum samples [63]. They also found anti-SaCas9 and anti-SpCas9 T-cells in 67% of donor serum and demonstrated a Cas9-specific cytokine response. As humans can have pre-existing adaptive immune responses to Cas9, this can pose as a risk during clinical trials when using CRISPR-based technologies to treat diseases. 

Fortunately, the eye is a relatively small and enclosed compartment, which allows for lower doses of therapeutics to be required for delivery, and a lower risk of dispersion to other tissues and organs [25]. The blood-retinal barrier is made up of tight junctions between the endothelial cells of retinal microvasculature and between the RPE cells, so the introduction of foreign material is less likely to escape the eye and cause an elevated inflammatory response. One recent study [64] looked specifically in the eye, and tested paired vitreous and serum samples for antibodies against SaCas9 and SpCas9 in patients undergoing vitreoretinal surgery. They found detectable α-Cas9 in serum samples, but no detectable α-Cas9 in the vitreous fluid, except in cases of prior bacterial ocular infection or damage to the blood-retina-barrier. This indicates that in humans, the intraocular presence of anti-Cas9 is low. While this data is encouraging for the future of CRISPR-Cas9 clinical trials in the eye, further research is needed to understand the extent of the functional consequences of delivery of these gene editing components. 

Lastly, gene editors are generally too large to fit into a single AAV for delivery, so studies use dual-AAV approaches [36,37,38]. In these approaches, the base editor is divided into two halves, an N-terminal and C-terminal half. Unfortunately, editing efficiency generally decreases with dual-AAV, due to the need for simultaneous transduction of multiple AAVs [37]. Recently, David Liu’s group published a paper [65] where they constructed small ABE8e variants to develop highly efficient single-AAV vectors to increase targeting capability in the heart, muscle, and liver. They identified the minimal components of the AAV genome, and were therefore able to package the base editor, the guide RNA, and all necessary promoters and regulatory sequences into a single AAV. With the single-AAV ABE construct, they saw further improvement in editing at a 33% and 22% editing efficiency in heart and muscle, respectively. This equated to a 2.1-fold and 2.5-fold increase in editing compared with the highest dose using dual-AAV treatment. This study shows tremendous promise for the use of base editing in disease, as it allows for an increase in efficiency while also bypassing the need to construct multiple AAV vectors.

## 13. Conclusions and Future Directions

To date, as this is a new technology being investigated in preclinical studies, there are no ongoing clinical trials in IRD patients using either base or prime editing. However, this technology holds potential for retinal monogenic disorders due to the increased editing efficiency, lack of double-stranded breaks in the DNA, and the ability for a permanent and stable genome edit. In the above studies, base and prime editing have been established as strong methods for prolonging visual function and retinal cell survival in preclinical models of IRDs. The rapid development of genome-editing technologies and their optimized variants, alongside continued efforts to increase editing efficiency and accessibility, will likely result in the publication of successful therapeutic testing in preclinical disease systems. Therefore, base and prime editing approaches have the potential to lead to the development of human clinical trials for treating IRDs, and the onset of therapeutic options for patients suffering from visual impairment.
